# Identification of cell division cycle 20 as a candidate biomarker and potential therapeutic target in bladder cancer using bioinformatics analysis

**DOI:** 10.1042/BSR20194429

**Published:** 2020-07-27

**Authors:** Peilin Shen, Xuejun He, Lin Lan, Yingkai Hong, Mingen Lin

**Affiliations:** 1 Department of Urology, The First Affiliated Hospital of Shantou University Medical College, Shantou, China; 2 The Second ward, Shantou University Mental Health Center, Shantou, China

**Keywords:** Bioinformatics, Biomarker, Bladder cancer, CDC20, therapeutics

## Abstract

**Purpose:** As bladder cancer (BC) is very heterogeneous and complicated in the genetic level, exploring genes to serve as biomarkers and therapeutic targets is practical.

**Materials and methods:** We searched Gene Expression Omnibus (GEO) and downloaded the eligible microarray datasets. After intersection analysis for identified differentially expressed genes (DEGs) of included datasets, overlapped DEGs were identified and subsequently analyzed with Gene Ontology (GO), Kyoto Encyclopedia of Genes and Genomes (KEGG), Protein–Protein Interaction (PPI) and hub genes identification. Hub genes were further analyzed with mRNA expression comparation in Oncomine and Gene Expression Profiling Interactive Analysis (GEPIA) database, proteomics-based validation in The Human Protein Atlas (THPA) and survival analysis in GEO and Oncolnc database.

**Results:** We analyzed five eligible GEO datasets and identified 76 overlapped DEGs mapped into PPI network with 459 edges which were mainly enriched in cell cycle pathway and related terms in GO and KEGG analysis. Among five identified hub genes, which are Cyclin-Dependent Kinase 1 (CDK1), Ubiquitin-Conjugating Enzyme E2 C (UBE2C), Cell Division Cycle 20 (CDC20), Microtubule Nucleation Factor (TPX2) and Cell Division Cycle Associated 8 (CDCA8); CDC20 and CDCA8 were confirmed as significant in mRNA expression comparation and proteomics-based validation. However, only CDC20 was considered prognostically significant in both GEO and Oncolnc database.

**Conclusions:** CDC20 and CDCA8 were identified as candidate diagnostic biomarkers for BC in the present study; however, only CDC20 was validated as prognostically valuable and may possibly serve as a candidate prognostic biomarker and potential therapeutic target. Still, further validation studies are essential and indispensable.

## Introduction

Bladder cancer (BC) is the ninth most common diagnostic cancer and the second most common diagnostic uro-oncological disease worldwide [1,2]. In 2018, 81190 people in the U.S.A. were diagnosed with BC, among which approximately 75% were non-muscle-invasive bladder cancer (NMIBC), and 25% were muscle-invasive bladder cancer (MIBC) [3,4]. Despite there are various treatment modalities, the prognosis of BC is still far from satisfactory even after appropriate therapy. In NMIBC, the recurrence rate is approximately 50–70%, and the progression rate is 1–2% in low-grade tumor and nearly 45% in high-grade tumor [5–7]. After progressing to MIBC, the 5-year survival rate is less than 50% [8,9]. As a tumor with lower grade and earlier stage usually has a better prognosis after treatment, early detection of BC is essential for the improvement of survival rate.

Even to this day, cystoscopy and urinary cytology are still the standard diagnostic and follow-up method for BC, however, both with some shortcomings. Cystoscopy is invasive and with the risk of a series of complications despite having high sensitivity and specificity. As an alternative to cystoscopy, although urinary cytology is noninvasive and highly specific, the overall sensitivity rate is only 33–48% [10,11]. Nowadays, many novel biomarkers for BC have emerged, but none of them has been confirmed as having reasonable sensitivity and specificity to be applied widely. Major guidelines of BC such as National Comprehensive Cancer Network (NCCN) guidelines [12], European Association of Urology (EAU) guidelines [13] and American Urological Association (AUA) guidelines [14] only recommend current biomarkers with low evidence strength. Furthermore, protein markers such as bladder tumor antigen (BTA) and nuclear matrix protein 22 (NMP22) reflect mainly an infection or inflammation rather than the oncologic characteristics of the tumor which may increase the misdiagnosis rate.

BC is a very heterogeneous and complicated disease at the genetic level. As it has been reported that BC is highly associated with multiple mRNA, long non-coding RNA (lncRNA) and miRNA, new genetic biomarkers may provide more important information than protein markers and serve as diagnostic and prognostic indicators [15]. Furthermore, genes with prognostic value may be involved with the mechanism of tumorigenesis and development which can be explored as therapeutic targets as well.

To help identify sufficient biomarker with diagnostic and prognostic values and potential therapeutic target for BC, we performed this integrated bioinformatics analysis using the data from Gene Expression Omnibus (GEO), Oncomine, Genotype-Tissue Expression (GTEx) project, The Human Protein Atlas (THPA) and The Cancer Genome Atlas (TCGA).

## Materials and methods

**Figure 1:**
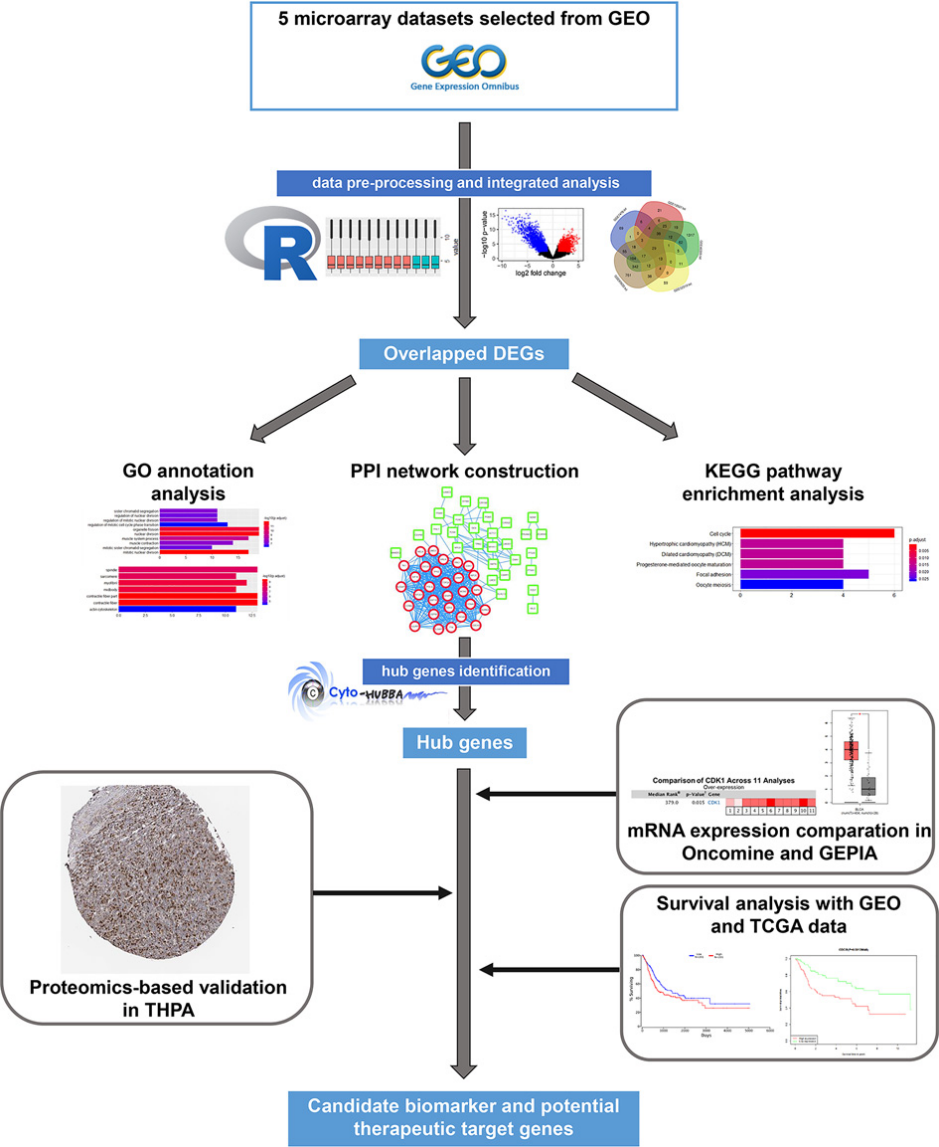
Flow chart of bioinformatics analysis Abbreviations: DEG, differentially expressed gene; GEPIA, Gene Expression Profiling Interactive Analysis; GO, Gene Ontology; KEGG, Kyoto Encyclopedia of Genes and Genomes; PPI, Protein–Protein Interaction.

The flow chart of integrated bioinformatics analysis in the presented study is shown in Figure 1. The full R code and generated expression matrixes of every GEO dataset we used are provided as supplementary materials in Supplementary Files S1 and S2.

### GEO microarray data pre-processing

We performed a search in the GEO database (https://www.ncbi.nlm.nih.gov/geo/) for candidate microarray datasets related to BC through 14 September 2019. The term ‘bladder cancer’ was used as a keyword for searching, and the organism was restricted in ‘*Homo sapiens*’. After carefully screening the retrieving results including various types of samples and expression data according to the inclusion and exclusion criteria, the eligible datasets were taken to the next step of data pre-processing. The inclusion criteria are as follows: (1) human bladder tumor samples versus normal bladder tissue samples; (2) mRNA expression profile; (3) available raw data. The exclusion criteria are as follows: (1) bladder tumor samples were restricted in a single pathological type; (2) raw data were unable to be processed due to technical issue; (3) sample size was less than 10.

All eligible datasets were pre-processed individually using R language software. Robust multi-array average (RMA) method [16] was used for background correcting, normalization and summarization. We considered datasets with high degree of inconsistency in the median, the upper and lower quartiles as with poor quality which would be ruled out. Annotation was performed using different annotation packages according to different platforms of the datasets.

### Integrated analysis of gene expression datasets

Eligible microarray datasets were further analyzed in R language. By using the *t* test method in the Linear Models for Microarray (LIMMA) package [17], we identified differentially expressed genes (DEGs) between tumor samples and normal bladder tissue samples with the cut-off criteria of fold change ≥ 2.0 and *P*<0.01. Subsequently, volcano plots were generated to demonstrate the results of DEGs selection using the ggplot2 package [18]. Finally, the intersection analysis was performed for the DEGs of all datasets using Venn diagram webtool (bioinformatics.psb.ugent.be/webtools/Venn/) to picked out the overlapped DEGs.

### Gene Ontology annotation analysis and pathway enrichment analysis

We used clusterProfiler package [19] in R language to conduct Gene Ontology (GO) [20] annotation analysis and Kyoto Encyclopedia of Genes and Genomes (KEGG) [21] pathway enrichment analysis for the overlapped DEGs. Adjusted *P*-value <0.05 and gene counts > 10 were considered as statistically significant in the three components of GO analysis, including biological process (BP), cellular component (CC) and molecular function (MF). As for KEGG analysis for biochemistry pathways, we set the cut-off criteria as adjusted *P*-value <0.05 to indicate a statistical significance.

### Protein–protein interaction network construction and hub genes identification

We established a protein–protein interaction (PPI) network by mapping the overlapped DEGs to the Search Tool for the Retrieval of Interacting Genes (STRING) database (version 10.5) (string-db.org) [22]. Interactive relationships among the overlapped DEGs were considered statistically significant with a combined score > 0.4. The result generated by the STRING database was input into Cytoscape software (version 3.6.1) [23] for visualization demonstration. CytoHubba (version 0.1) [24], a plugin Cytoscape was used to identify the hub genes which were defined as the top five ranked genes according to the connectivity degree levels of each protein node.

### Assessment of hub genes in Oncomine and Gene Expression Profiling Interactive Analysis database

The identified hub genes were assessed through comparative analysis of mRNA in Oncomine (version 4.5) (www.oncomine.org) [25] and Gene Expression Profiling Interactive Analysis (GEPIA; gepia.cancer-pku.cn/index.html) [26]. Oncomine database is a publicly accessible online data-mining platform containing various cancer microarray data and provides multiple kind of integrated analysis of gene expression. GEPIA is a web tool for RNA sequencing expression data analysis of 9736 tumors and 8587 normal samples from the TCGA database and the GTEx projects. Comparative analysis of each hub gene was performed individually in two databases. In Oncomine, the analysis type was restricted in ‘Bladder Cancer vs. Normal Analysis’ and the data type was restricted in ‘mRNA’. After the comparison of hub gene expression between BC samples and normal bladder tissue samples across included analyses, a comparison figure would be generated by the database automatically demonstrating the median rank and combined P value of each hub gene. As for GEPIA, we used box-plots to compare the mRNA expression of hub genes between TCGA bladder tumors vs TCGA normal bladder tissues + GTEx normal bladder tissues with the cut-off criteria of |Log2FC| > 1 and *P*-value <0.01. Those with significant overexpression in BC in both databases were taken to further proteomics-based validation.

### Proteomics-based validation of hub genes in THPA

THPA [27] contains immunohistochemistry images showing antibody staining in samples from 144 individuals corresponding to 44 different normal tissue types, and samples from 216 cancer patients corresponding to 20 different types of cancer by using tissue microarrays technology. We downloaded the histological section images and corresponding information of significantly overexpressed hub genes from normal urinary bladder tissues and urothelial cancer tissues of bladder obtained by immunohistochemistry in THPA. Because the antibody staining is already reported as not detected, low, medium or high based on the staining intensity and fraction of stained cells, we conducted the Mann–Whitney Test in SPSS 19.0 to compare the antibody staining level of hub genes between normal urinary bladder tissues and urothelial cancer tissues of bladder. The cutoff *P*-value was set as 0.05 and the hub genes with significant antibody staining in urothelial cancer cells were considered as significant hub genes which may be useful for BC diagnosis.

### Survival analysis of hub genes using GEO and TCGA data

In order to determine the association between hub genes and clinical outcomes, significant hub genes validated by mRNA comparative analysis and proteomics-based validation were taken to further survival analysis. Expression and survival data from GEO were analyzed in R language software with the survival package [28], while survival plots were automatically generated in the online database Oncolnc (www.oncolnc.org) which is based on TCGA data and contains both RNA expression data and survival data of up to 21 different kinds of cancers [29]. A hub gene with *P*<0.05 was considered as a prognostically valuable gene, the high expression of which was significantly related to poor prognosis; therefore, it can be regarded as a candidate prognostic biomarker or potential therapeutic target for BC.

## Results

### Identification of DEGs in BC

**Figure 2:**
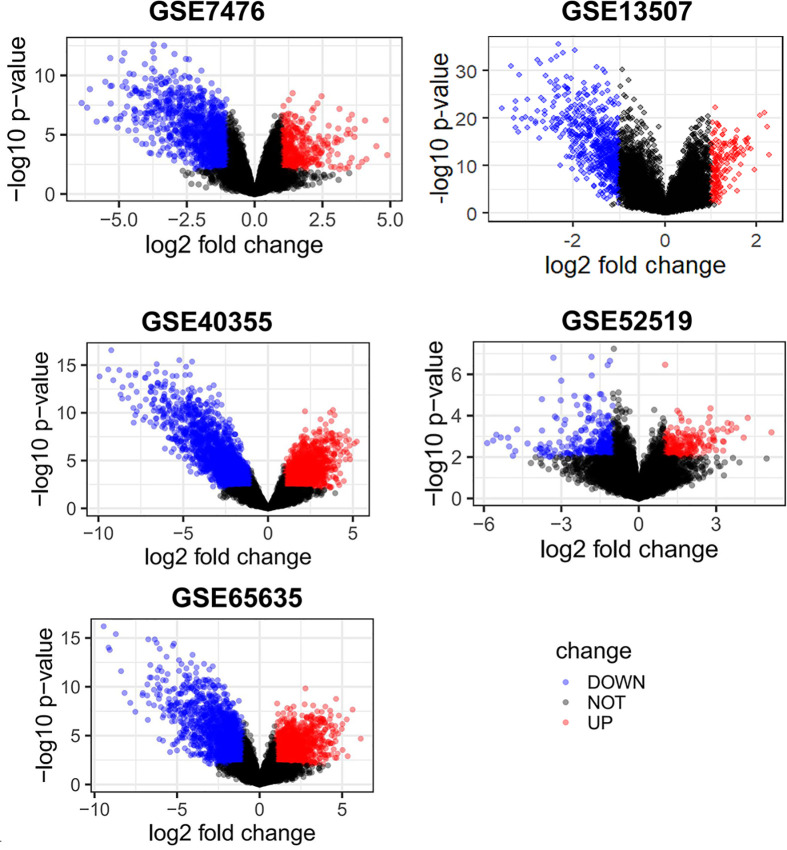
Identified DEGs of included datasets DEGs between BC samples and normal bladder tissue samples were identified with the cut-off criteria of fold change ≥ 2.0 and *P*<0.01. The up-regulated DEGs were 425 in GSE7476, 182 in GSE13507, 2008 in GSE40355, 211 in GSE52519 and 1470 in GSE65635. The down-regulated DEGs were 1233 in GSE7476, 634 in GSE13507, 2510 in GSE40355, 315 in GSE52519 and 1761 in GSE65635.

**Figure 3:**
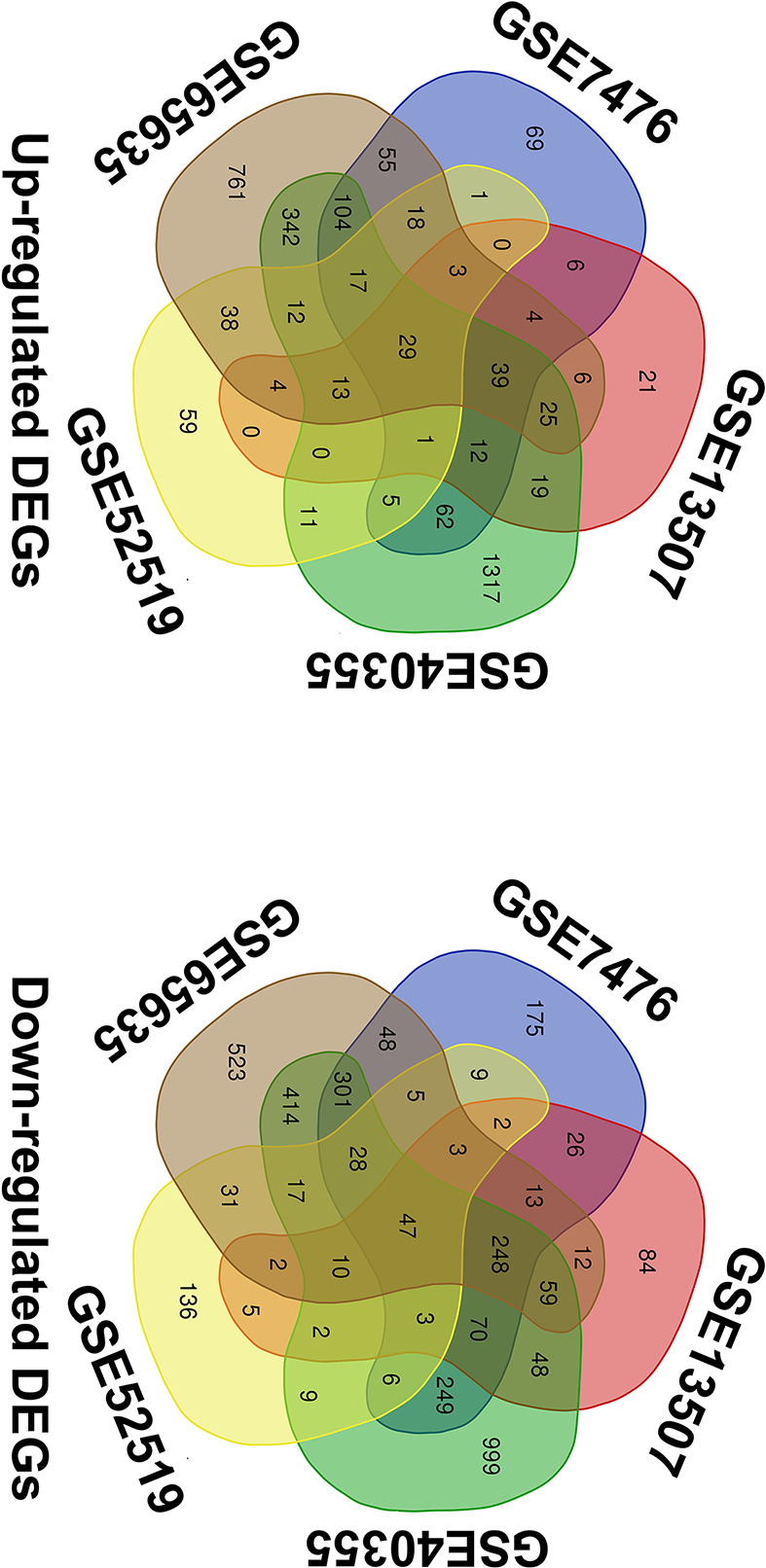
Overlapped DEGs from the intersection analysis of included datasets DEGs of GSE7476, GSE13507, GSE40355, GSE52519 and GSE65635 were input into Venn diagram webtool for intersection analysis and 76 overlapped DEGs with 29 up-regulated genes and 47 down-regulated genes were identified.

With the search of GEO database, 4795 results were found for screening, in which 7 microarray datasets including GSE7476, GSE13507, GSE31189, GSE40355, GSE52519, GSE65635 and GSE121711 appeared to be eligible according to inclusion and excluding criteria. After pre-processing, the results of all datasets showed superior quality except for the result of GSE31189 and GSE121711, and therefore excluded for further analysis (Supplementary File S3). The remaining five datasets are all with a sample size > 10 and the platforms utilized by different datasets were varied (Table 1). By screening the expressed genes with the cut-off criteria, the DEGs were identified for each dataset. The up-regulated DEGs were 425 in GSE7476, 182 in GSE13507, 2008 in GSE40355, 211 in GSE52519 and 1470 in GSE65635. The down-regulated DEGs were 1233 in GSE7476, 634 in GSE13507, 2510 in GSE40355, 315 in GSE52519 and 1761 in GSE65635 (Figure 2). Subsequently, 29 up-regulated and 47 down-regulated overlapped DEGs were calculated using intersection analysis (Figure 3).

**Table 1:** Characteristics of included datasets

Dataset	Platform	Bladder cancer	Normal
GSE7476	GPL570 Affymetrix Human Genome U133 Plus 2.0 Array	9	3
GSE13507	GPL6102 Illumina human-6 v2.0 expression beadchip	188	67
GSE40355	GPL13497 Agilent-026652 Whole Human Genome Microarray 4x44K v2	16	8
GSE52519	GPL6884 Illumina HumanWG-6 v3.0 expression beadchip	9	3
GSE65635	GPL14951 Illumina HumanHT-12 WG-DASL V4.0 R2 expression beadchip	8	4

### Functional enrichment analysis

**Figure 4:**
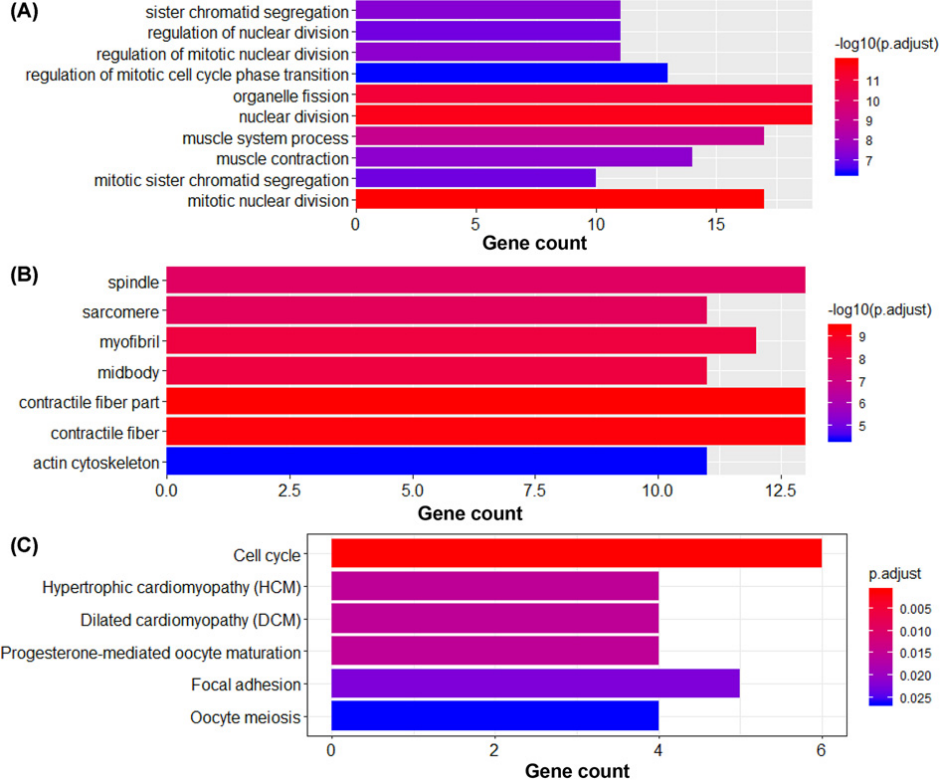
GO annotation analysis and KEGG pathway enrichment analysis of overlapped DEGs (**A**) Terms enriched in biological processes of GO enrichment analysis were as follows: mitotic nuclear division, nuclear division, organelle fission, muscle system process, muscle contraction, regulation of mitotic nuclear division, sister chromatid segregation, regulation of nuclear division, regulation of mitotic cell cycle phase transition, chromosome segregation, nuclear chromosome segregation, regulation of cell cycle phase transition, muscle cell differentiation. (**B**) Terms enriched in CCs of GO enrichment analysis were as follows: contractile fiber part, contractile fiber, myofibril, midbody, sarcomere, spindle, actin cytoskeleton. (**C**) Pathways enriched in KEGG analysis were Cell cycle, Hypertrophic cardiomyopathy (HCM), Dilated cardiomyopathy (DCM), Progesterone-mediated oocyte maturation, Focal adhesion and Oocyte meiosis.

All 76 overlapped DEGs were processed with GO and KEGG analysis. For GO analysis, BP was the most favorable enrichment component, in which DEGs were significantly enriched in mitotic nuclear division, nuclear division, organelle fission and so on (Figure 4A). CC analysis was enriched in contractile fiber part, contractile fiber, myofibril, etc (Figure 4B). For MF analysis, although there were significantly enriched terms, none of them contained more than ten DEGs. In addition, the results of KEGG pathway enrichment analysis indicated that DEGs were most enriched in cell cycle pathway (Figure 4C).

### PPI network and hub genes

**Figure 5:**
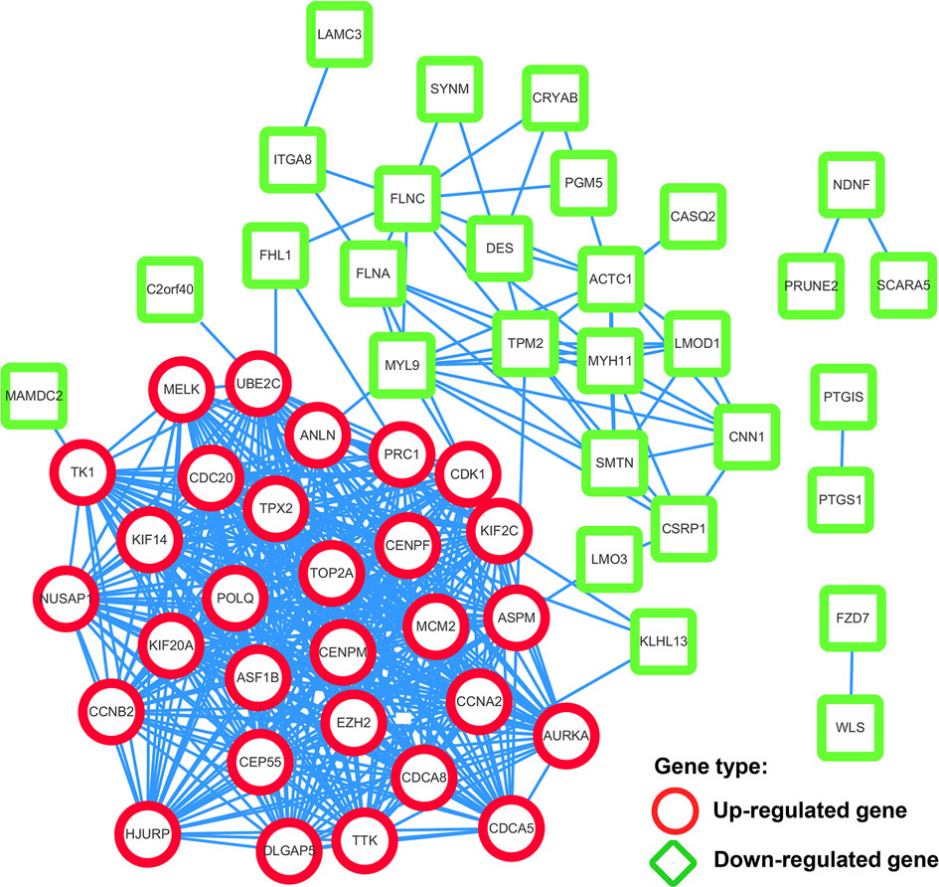
PPI network of the overlapped DEGs The PPI network contains 58 nodes and 459 edges which roughly clustered into two sets depending on the types of genes were up-regulated or down-regulated.

PPI network was constructed with STRING tool and visualized with Cytoscape software. There were 76 nodes and 459 edges in the PPI network generated by STRING tool originally. After removing 18 unconnected nodes, the network with 58 nodes and 459 edges was visualized in Cytoscape, which roughly clustered into two sets depending on the types of genes were up-regulated or down-regulated (Figure 5). In addition, the PPI enrichment *P*-value is less than 1.0E-16 which reflected significant interactions among the overlapped DEGs. The calculated top five hub genes according to connectivity degree were all up-regulated genes and ranked in the sequence of Cyclin-Dependent Kinase 1 (CDK1), Ubiquitin-Conjugating Enzyme E2 C (UBE2C), Cell Division Cycle 20 (CDC20), Microtubule Nucleation Factor (TPX2) and Cell Division Cycle Associated 8 (CDCA8) (Table 2).

**Table 2:** Top five hub genes with the highest degree of connectivity

Rank	Gene symbol	Gene description	Connectivity degree
1	CDK1	Cyclin-Dependent Kinase 1	30
1	UBE2C	Ubiquitin-Conjugating Enzyme E2 C	30
3	CDC20	Cell Division Cycle 20	29
3	TPX2	Microtubule Nucleation Factor	29
3	CDCA8	Cell Division Cycle Associated 8	29

### Comparative mRNA expression analysis of hub genes

**Figure 6:**
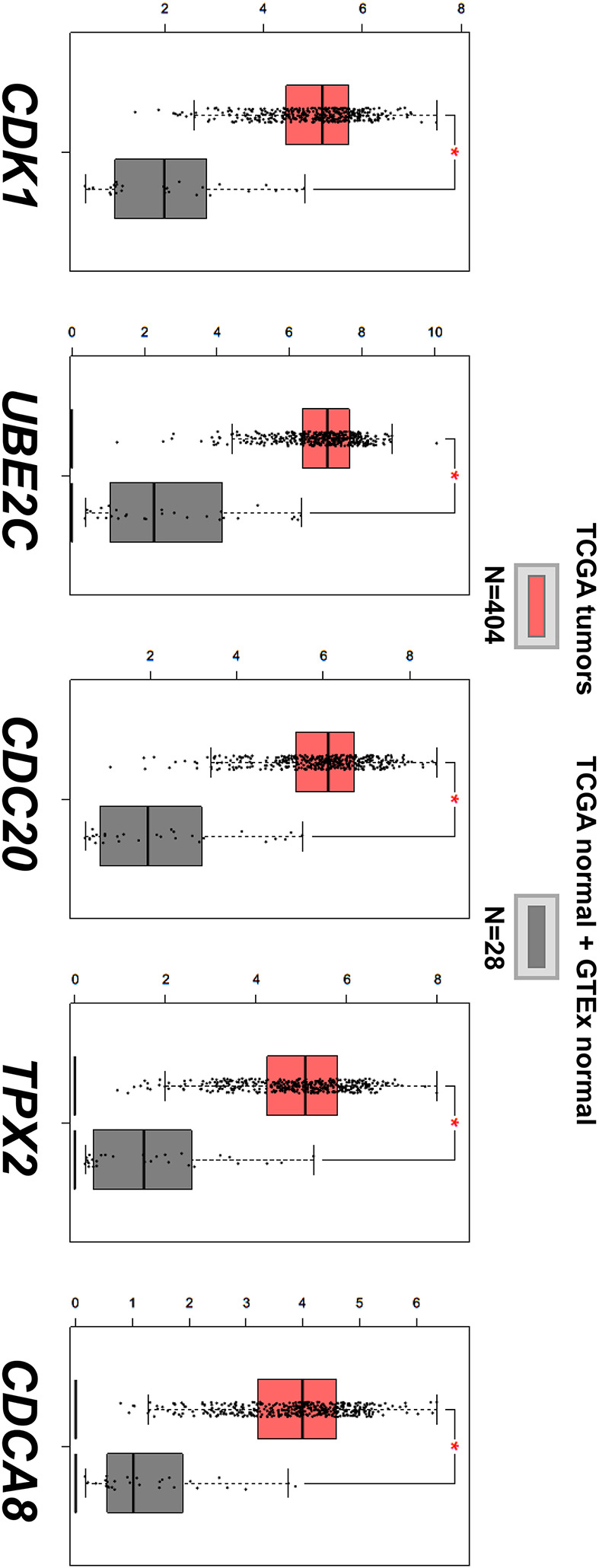
Box-plots of hub genes mRNA expression between BC and normal bladder in GEPIA database We compared the mRNA expression of CDK1, UBE2C, CDC20, TPX2 and CDCA8 between BC and normal bladder in GEPIA database using data from TCGA and GTEx. We considered |Log2FC| > 1 and *P*-value <0.01 as significant and all five genes met the statistical standard.

**Figure 7:**
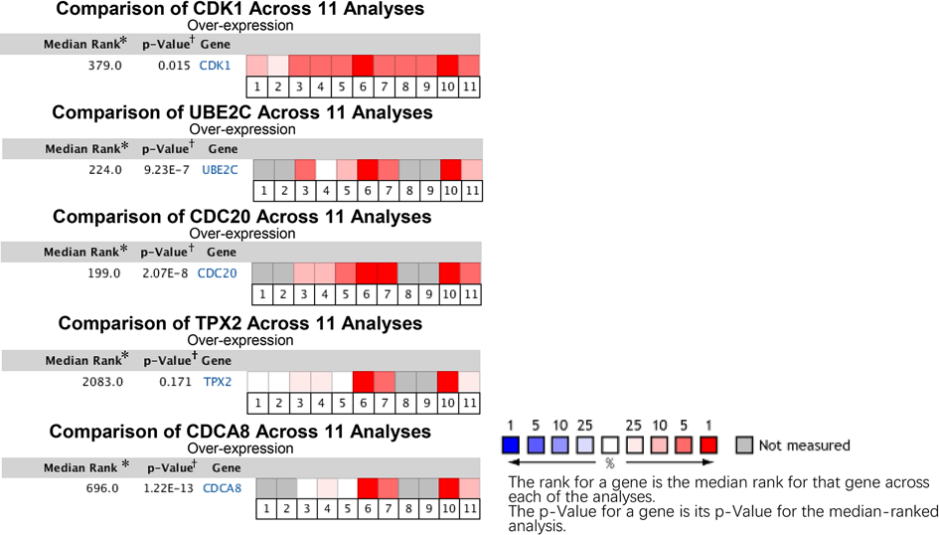
Pooled comparative analyses on the mRNA expression of hub genes in Oncomine database * The rank for a gene is the median rank for that gene across each of the analyses. † The *P*-value for a gene is its *P*-value for the median-ranked analysis. 1 Infiltrating Bladder Urothelial Carcinoma vs. Normal (Blaveri Bladder 2, *Clin. Cancer Res.*, 2005). 2 Superficial Bladder Cancer vs. Normal (Blaveri Bladder 2, *Clin. Cancer Res.*, 2005). 3 Infiltrating Bladder Urothelial Carcinoma vs. Normal (Dyrskjot Bladder 3, *Cancer Res.*, 2004). 4 Stage 0 is Bladder Urothelial Carcinoma vs. Normal (Dyrskjot Bladder 3, *Cancer Res.*, 2004). 5 Superficial Bladder Cancer vs. Normal (Dyrskjot Bladder 3, *Cancer Res.*, 2004). 6 Infiltrating Bladder Urothelial Carcinoma vs. Normal (Lee Bladder, *J. Clin. Oncol.*, 2010). 7 Superficial Bladder Cancer vs. Normal (Lee Bladder, *J. Clin. Oncol.*, 2010). 8 Infiltrating Bladder Urothelial Carcinoma vs. Normal (Modlich Bladder, *Clin. Cancer Res.*, 2004). 9 Superficial Bladder Cancer vs. Normal (Modlich Bladder, *Clin. Cancer Res.*, 2004). 10 Infiltrating Bladder Urothelial Carcinoma vs. Normal (Sanchez-Carbayo Bladder 2, *J. Clin. Oncol.*, 2006). 11 Superficial Bladder Cancer vs. Normal (Sanchez-Carbayo Bladder 2, *J. Clin. Oncol.*, 2006).

Identified hub genes were assessed with comparative pooled analysis using Oncomine and GEPIA database. All five hub genes were significantly overexpressed in BC samples in GEPIA database (Figure 6). In Oncomine database, only TPX2 (*P*=0.171) showed a statistically insignificant difference when comparing the gene expression between BC sample and normal bladder tissue samples while CDK1 (*P*=0.015), UBE2C (*P*=9.29E-7), CDC20 (*P*=2.07E-8) and CDCA8 (*P*=1.22E-13) were validated as significant (Figure 7). CDK1, UBE2C, CDC20 and CDCA8 showing significant higher expression in BC samples in both databases were taken to further proteomics-based validation.

### Proteomics-based validation of hub genes

**Figure 8:**
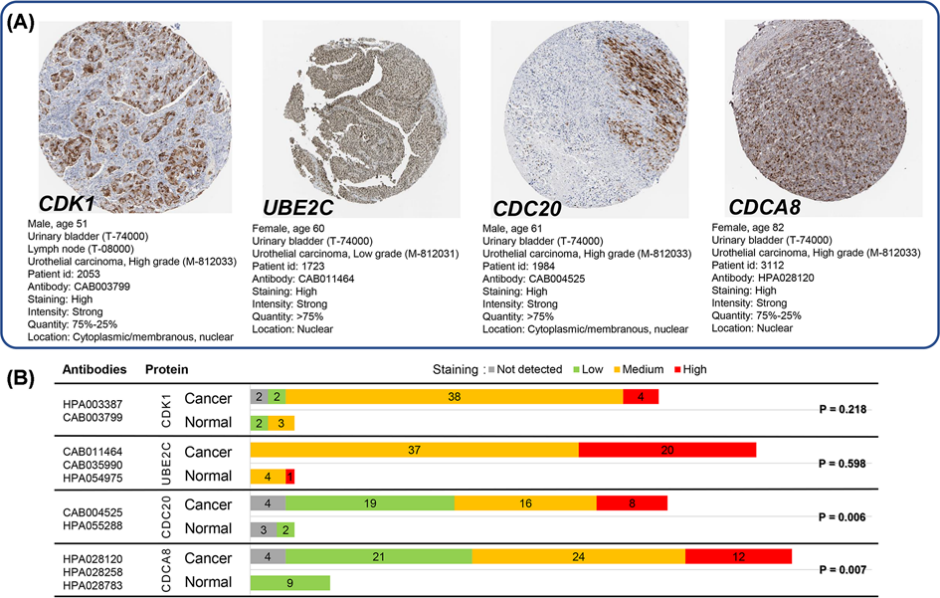
Antibody staining of hub genes in immunohistochemistry images between normal urinary bladder and urothelial cancer tissues of bladder (**A**) High antibody staining immunohistochemistry images of four hub genes are available at https://www.proteinatlas.org/ENSG00000170312-CDK1/pathology/urothelial+cancer# (CDK1), https://www.proteinatlas.org/ENSG00000175063-UBE2C/pathology/urothelial+cancer# (UBE2C), https://www.proteinatlas.org/ENSG00000117399-CDC20/pathology/urothelial+cancer# (CDC20) and https://www.proteinatlas.org/ENSG00000134690-CDCA8/pathology/urothelial+cancer# (CDCA8) in THPA, respectively. (**B**) Two antibodies were used for CDK1 (HPA003387 and CAB003799) and CDC20 (CAB004525 and HPA055288) and three for UBE2C (CAB011464, CAB035990 and HPA054975) and CDCA8 (HPA028120, HPA028258 and HPA028783). High positive staining rate was detected in urothelial cancer tissues of bladder for CDK1 (44/46), UBE2C (57/57), CDC20 (43/47) and CDCA8 (57/61), however, only CDC20 (*P*=0.006) and CDCA8 (*P*=0.007) showed significant results by comparing normal urinary bladder with urothelial cancer tissues of bladder using Mann–Whitney test. The cutoff *P*-value was set as 0.05.

We used THPA database to further verify the significance of CDK1, UBE2C, CDC20 and CDCA8 in protein level with immunohistochemistry images. High antibody staining images of all four genes can be found on THPA (Figure 8A) and high positive staining rate was detected in urothelial cancer tissues of bladder for CDK1 (44/46), UBE2C (57/57), CDC20 (43/47) and CDCA8 (57/61). However, only CDC20 (*P*=0.006) and CDCA8 (*P*=0.007) showed statistically significant results by comparing normal urinary bladder with urothelial cancer tissues of bladder using Mann–Whitney test, and can be considered as significant hub genes which may be useful for BC diagnosis (Figure 8B).

### Survival analysis of significant hub genes

**Figure 9:**
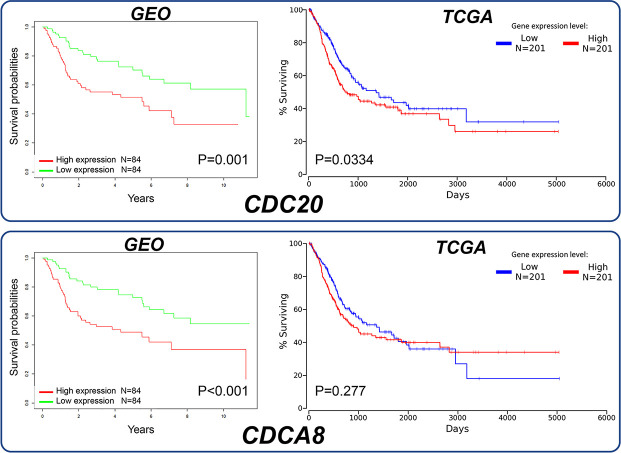
Survival analysis of significant hub genes We conducted survival analysis using GEO data with R language software and TCGA data in Oncolnc database to demonstrate the association between the expression of significant hub genes and overall survival time. CDC20 showed a statistical significance in both the databases (*P*=0.001 in GEO; *P*=0.0361 in Oncolnc), while CDCA8 only showed a statistical significance in GEO (*P*<0.001 in GEO; *P*=0.277 in Oncolnc).

The survival analysis was conducted for two significant hub genes. According to the survival plots in both the databases, high expression of CDC20 level was significantly associated with poor overall survival (*P*=0.001 in GEO; *P*=0.0361 in Oncolnc), which suggested that CDC20 may contribute to the mortality of BC patients. On the other hand, CDCA8 only showed a statistical significance in GEO (*P*<0.001 in GEO; *P*=0.277 in Oncolnc) which indicated that it is less possible to be further explored as a candidate prognostic biomarker or potential therapeutic target for BC (Figure 9).

## Discussion

As one of the most common diagnostic urologic tumors, it has been reported that BC has great heterogeneity and complexity at the molecular level. Therefore, exploring deep into the genetic level with integrated bioinformatics analysis, we tried to discover critical genes which can act as novel biomarkers with diagnostic and prognostic value or potential therapeutic targets for BC.

We searched the GEO database and selected five datasets for inclusion. By using R language software and Venn diagram webtool, 76 DEGs with 29 up-regulated genes and 47 down-regulated genes were identified. After GO annotation analysis and KEGG pathway enrichment analysis, DEGs were mostly enriched in terms and pathways related to cell cycle which play an important role in cell growth, anabolism and proliferation [30]. Precise cell proliferation and DNA replication are essential for maintaining genomic stability and uncontrolled cell cycle procedure is closely associated with tumor genesis and development, including BC [31,32]. Then, a PPI network with 76 nodes and 459 edges were constructed to better demonstrate the connection among identified DEGs and five hub genes were found with top connectivity degree, including CDK1, UBE2C, CDC20, TPX2 and CDCA8. The expression of each hub gene between BC and normal bladder tissues was assessed in Oncomine and GEPIA database individually. Except for TPX2, four other hub genes with significant overexpression in BC sample in both Oncomine and GEPIA were taken to further proteomics-based validation. By analyzing the antibody staining immunohistochemistry images of CDK1, UBE2C, CDC20 and CDCA8 in THPA using Mann–Whitney test, we considered CDC20 and CDCA8 as significant hub genes with diagnostic value for BC. To further analyze the value of significant hub genes, survival plots were generated using GEO and Oncolnc database. Unfortunately, only CDC20 showed a significant *P*-value in both databases and was regarded as a cancer-promoting gene which can be further explored as a prognostic biomarker or therapeutic target for BC.

CDCA8, a part of the chromosomal passenger complex (CPC), plays an important role in the cell division cycle in cancer cells [33]. There have been studies which argued that CDCA8 is a required initiating factor for the oncogenesis and progression of tumors [34]. However, researches about its role in BC is still limited and there is only one study shows that the overexpression of CDCA8 was found in BC and correlated with poor clinicopathological features of BC patients [35].

CDC20 is the only identified hub gene significantly associated with the diagnosis and prognosis of BC in the present study. In cell cycle progression, CDC20 encodes a protein functions as an essential regulator for cell division, the most important function of which is to combine with anaphase promoting complex (APC/C), and subsequently regulate the degradation of securin. Securin destruction promotes the degradation of cohesion, the separation of sister chromatid, and subsequently the transition from G_2_/M phase to G_1_ phase. In this case, normal cell cycle can be maintained. The dysfunction of CDC20 can contribute to poor differentiation, tumor aneuploidy and poor prognosis in multiple cancer, including BC [36]. Besides, it has been revealed that its overexpression can decrease overall survival time and recurrence-free survival time in BC patients, and even lead to progression [36]. Even so, its molecular mechanism is still not very clear, and researches concerning its association with BC is very limited. Therefore, CDC20 is with great potential to be further explored as a candidate biomarker and potential cancer therapeutic target in BC [37].

As an integrated bioinformatics analysis study, several limitations were inevitable. First, the results of the present study were calculated through multiple statistical steps. Therefore, by setting different cut-off criteria, applying different statistical method or using different tools, the results would be various correspondingly. Second, the inevitable heterogeneity among different datasets would impact on the reliability of the presented study. Third, no *in vitro* or *in vivo* experiment were designed for validation so that the conclusion is still theoretical and further experimental test confirmations are absolutely necessary.

In conclusion, two multiple cancer-associated genes including CDC20 and CDCA8 were identified as candidate diagnostic biomarkers for BC by analyzing GEO, Oncomine, GTEx, THPA and TCGA data. However, only CDC20 was validated as having prognostic value and may even serve as a candidate prognostic biomarker and potential therapeutic target for BC. For CDC20 and CDCA8, either as a biomarker or a therapeutic target, further validation research is still indispensable to confirm their clinical effect in the future, and we look forward to seeing more bioinformatics and experimental studies with larger sample size and more detailed clinical information to be carried out for the remediation and extension of our study. Hopefully, by exploring deep into CDC20, it can help move the diagnosis and therapy process of BC closer to consummation.

## Supplementary Material

Supplementary Materials Files S1-S3

## Data Availability

The data analyzed for the present study can be found in the GEO database (www.ncbi.nlm.nih.gov/geo/), Oncomine database (www.oncomine.org), GEPIA database (gepia.cancer-pku.cn/index.html), THPA database (www.proteinatlas.org) and Oncolnc database (www.oncolnc.org). The full R code and generated expression matrixes of every GEO dataset we used were provided as supplementary materials in Supplementary Files S1 and S2.

## Abbreviations

AUA: American Urological Association
CC: cellular component
CDC20: cell division cycle 20
CDCA8: cell division cycle associated 8
CDK1: cyclin-dependent kinase 1
DEG: differentially expressed gene
EAU: European Association of Urology
GEO: Gene Expression Omnibus
GEPIA: Gene Expression Profiling Interactive Analysis
GO: gene ontology
GTEx: Genotype-Tissue Expression
KEGG: Kyoto Encyclopedia of Genes and Genomes
lncRNA: long non-coding RNA
MF: molecular function
MIBC: muscle-invasive bladder cancer
NCCN: National Comprehensive Cancer Network
NMIBC: non-MIBC
PPI: protein–protein interaction
STRING: Search Tool for the Retrieval of Interacting Genes
TCGA: The Cancer Genome Atlas
THPA: The Human Protein Atlas
TPX2: microtubule nucleation factor
UBE2: Ubiquitin-Conjugating Enzyme E2 C
