# Supplementary figures and images for: Identification of cell division cycle 20 as a candidate biomarker and potential therapeutic target in bladder cancer using bioinformatics analysis
Source: Biosci Rep. 2020 Jul 27;40(7):BSR20194429. doi: 10.1042/BSR20194429 (PMC7385587; doi:10.1042/BSR20194429)

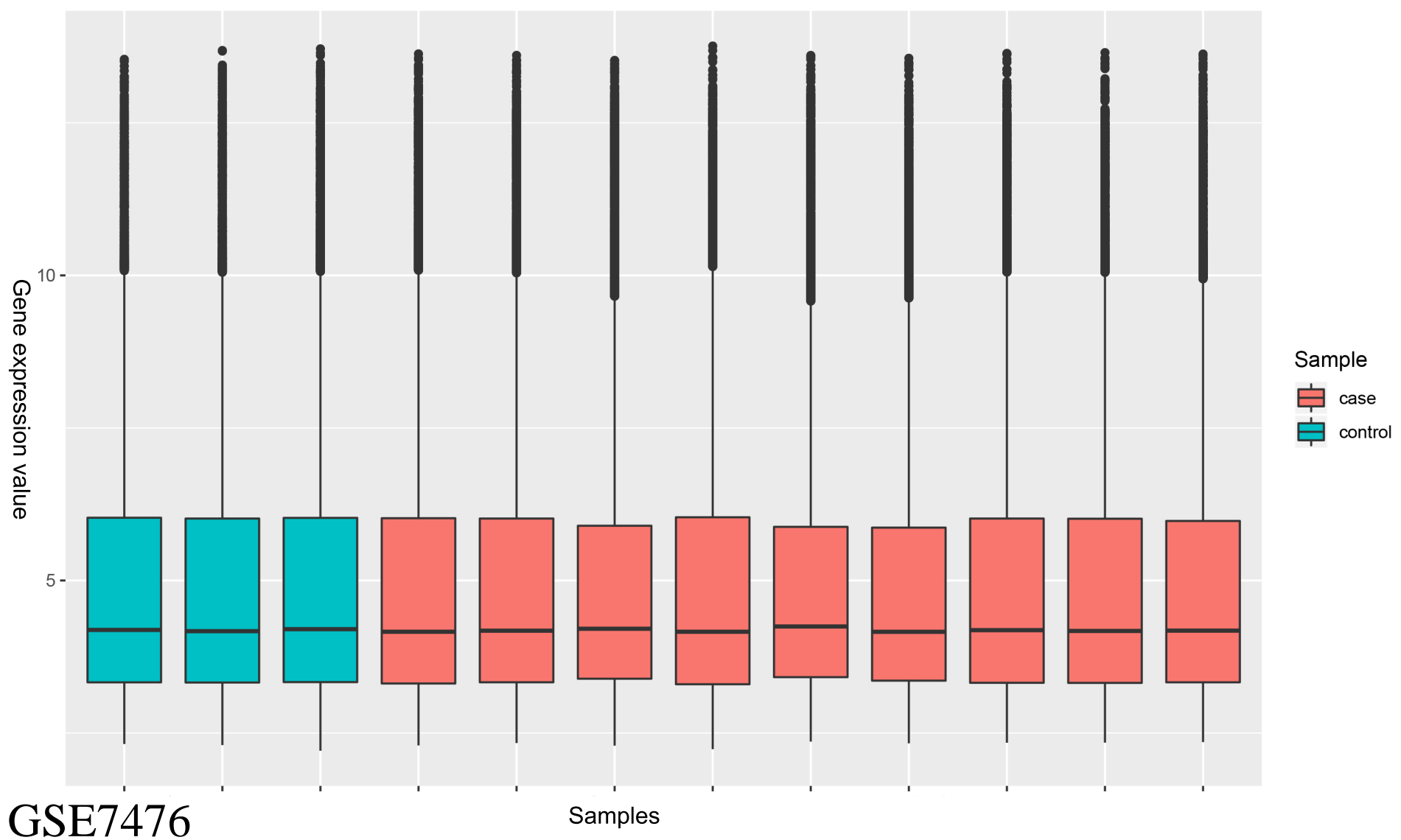

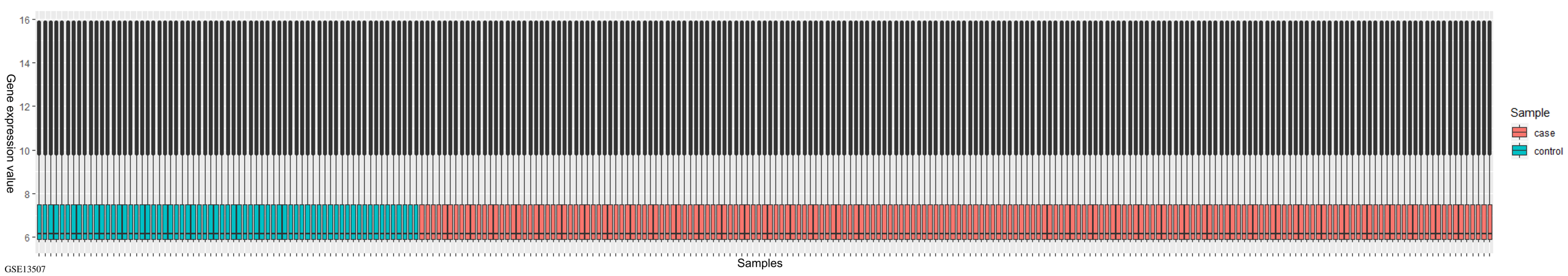

Gene expression value

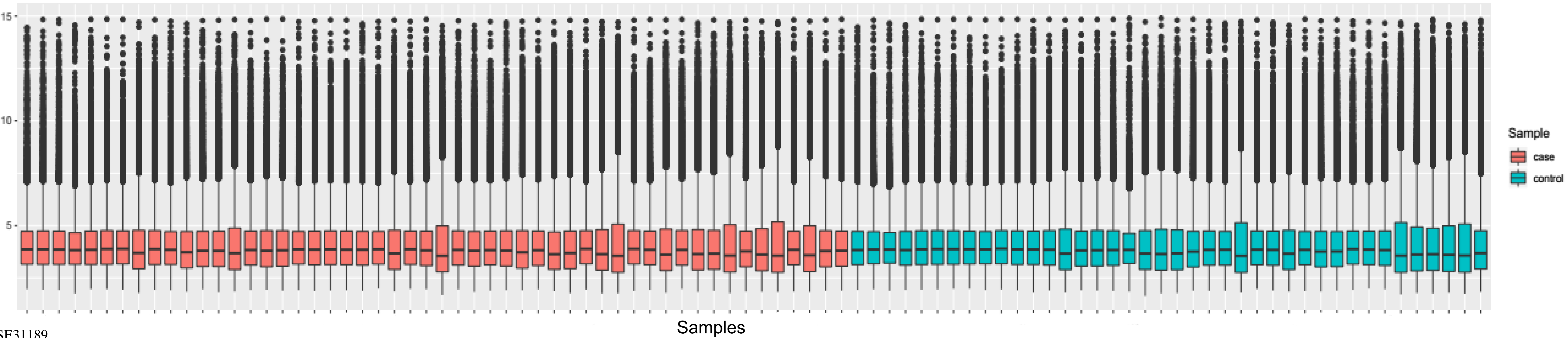

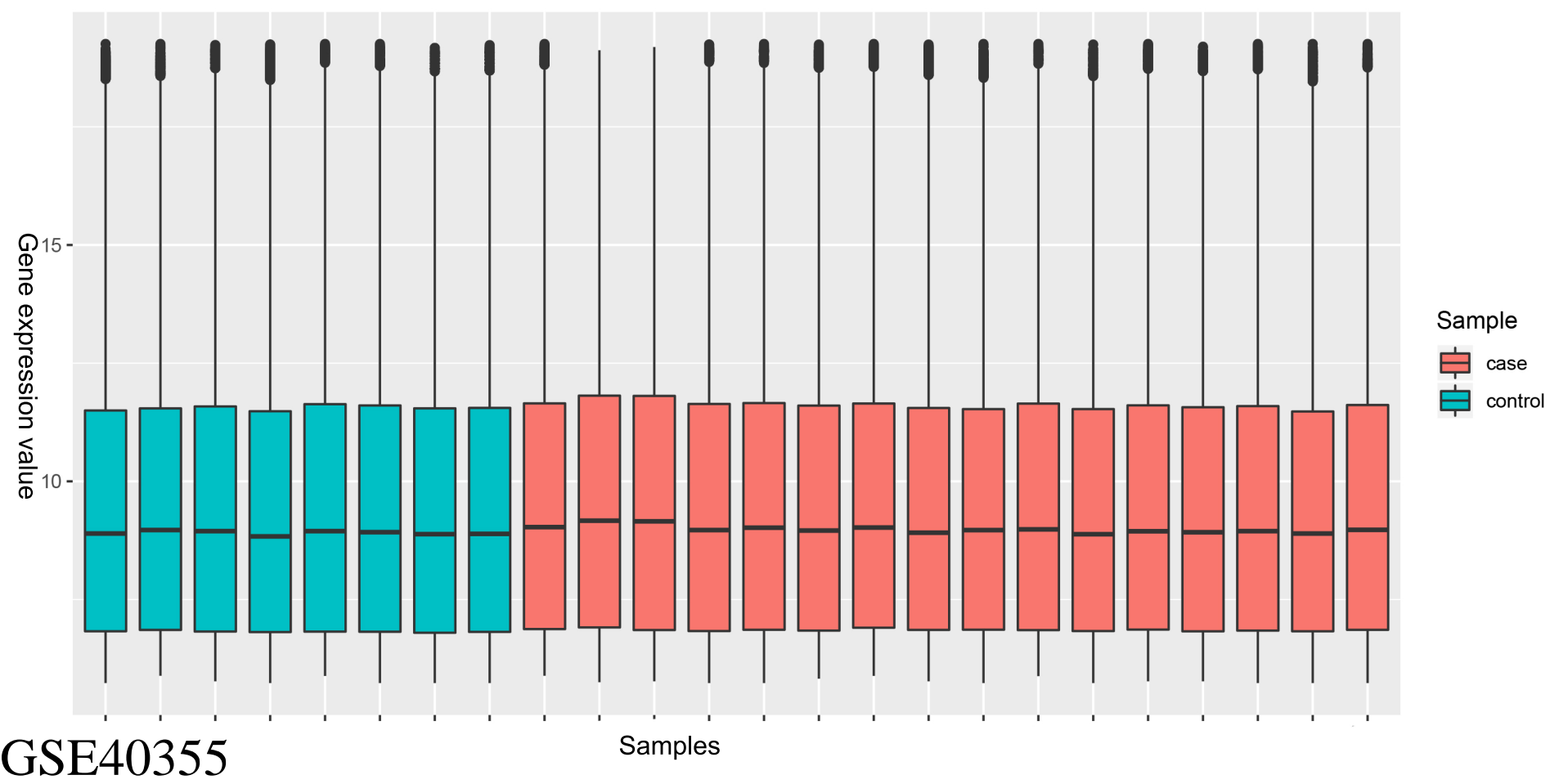

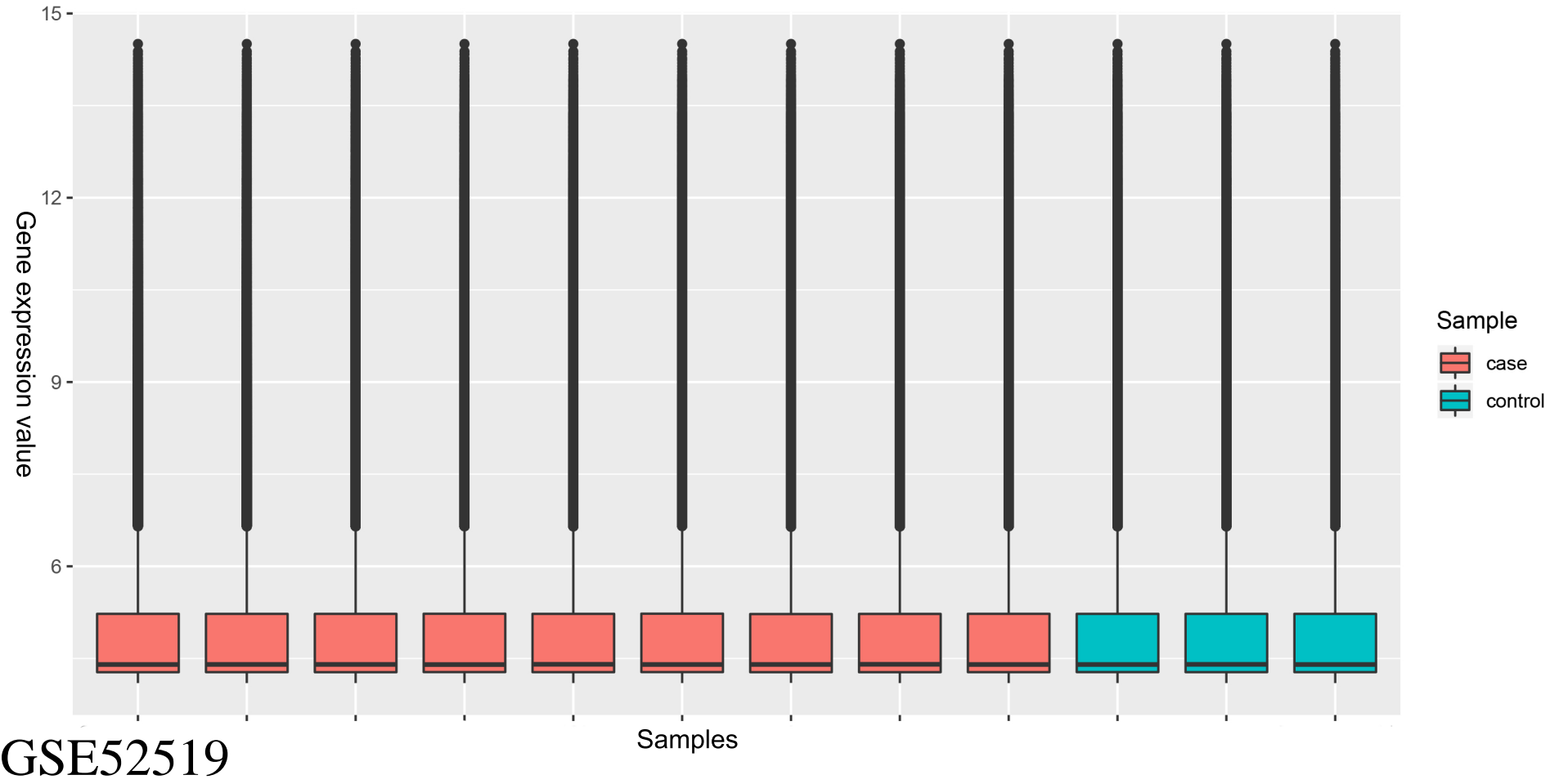

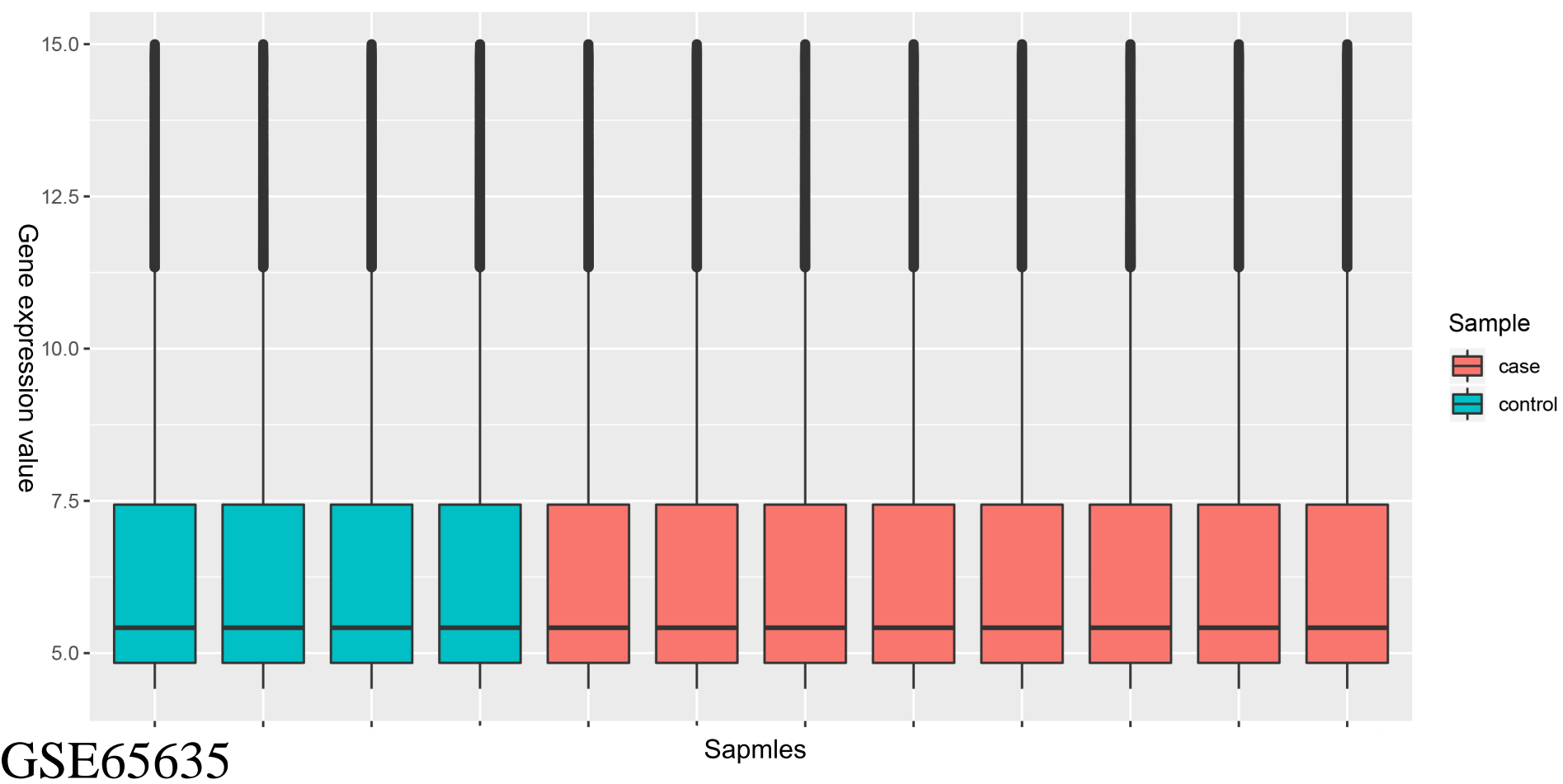

Sample

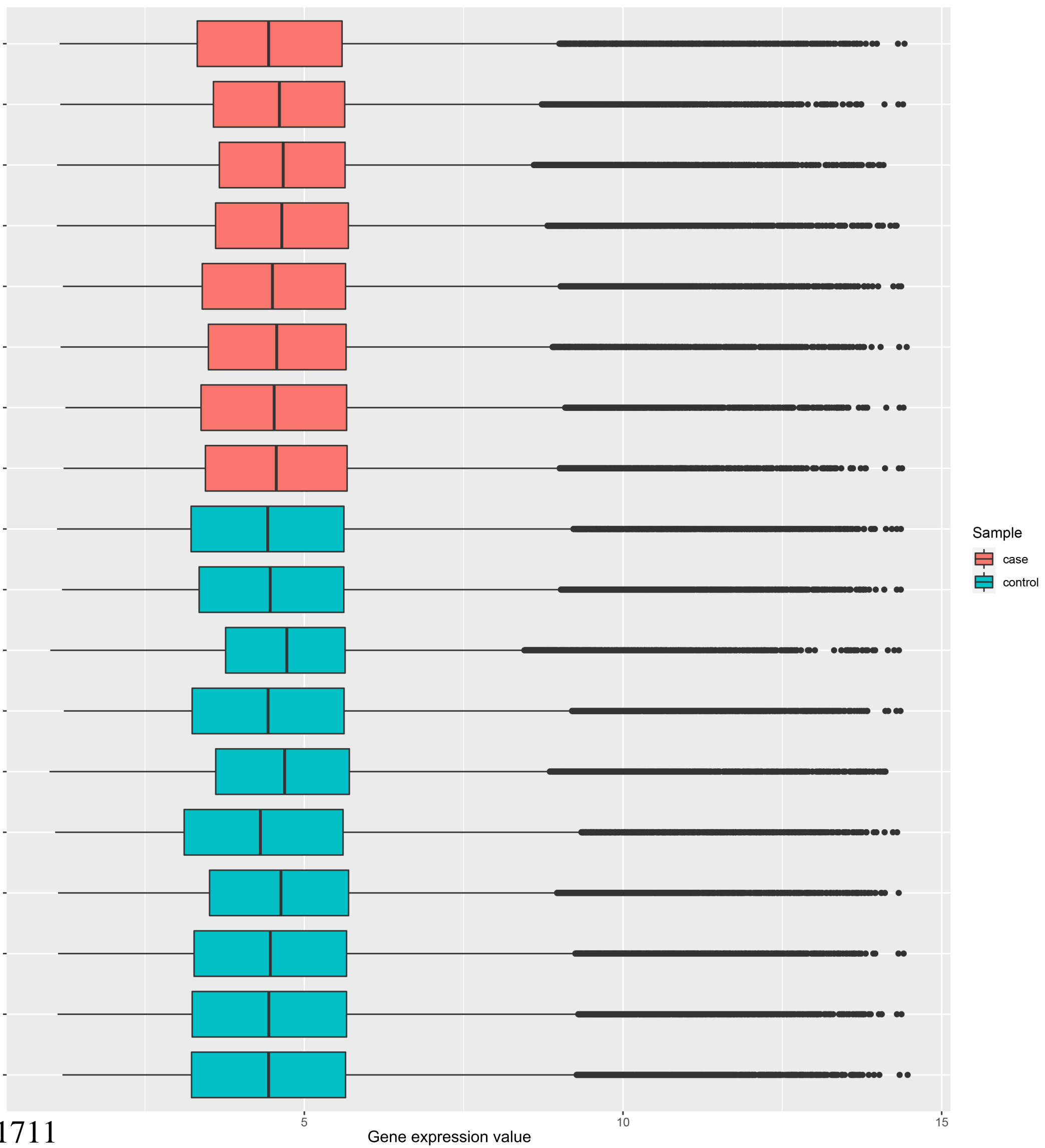

Supplement: Supplementary Materials Files S1-S3 [file BSR-2019-4429_supp.zip › BSR-2019-4429_suppS3.pdf]
